# Avoidance of surgery for head and neck infantile myofibromatosis using imatinib monotherapy

**DOI:** 10.1002/ccr3.5382

**Published:** 2022-02-09

**Authors:** Prasanth Pattisapu, Tara L. Wenger, John P. Dahl, Randall A. Bly, Juliana Bonilla‐Velez, Natalie Wu, Anurekha Hall, Erin R. Rudzinski, Jonathan A. Perkins

**Affiliations:** ^1^ Department of Otolaryngology–Head & Neck Surgery Nationwide Children's Hospital and The Ohio State University Columbus Ohio USA; ^2^ Center for Surgical Outcomes Research Abigail Wexner Research Institute Nationwide Children's Hospital Columbus Ohio USA; ^3^ 12353 Department of Genetic Medicine University of Washington School of Medicine Seattle Washington USA; ^4^ 12353 Department of Otolaryngology–Head & Neck Surgery University of Washington School of Medicine Seattle Washington USA; ^5^ 7274 Division of Pediatric Otolaryngology–Head & Neck Surgery Seattle Children's Hospital Seattle Washington USA; ^6^ 12353 Department of Pediatrics Division of Hematology/Oncology University of Washington School of Medicine Seattle Washington USA; ^7^ 7274 Cancer and Blood Disorders Center Seattle Children's Hospital Seattle Washington USA; ^8^ 7274 Division of Pathology Seattle Children's Hospital Seattle Washington USA; ^9^ 12353 Department of Pathology University of Washington School of Medicine Seattle Washington USA

**Keywords:** imatinib, Infantile myofibromatosis, PDGFRB, precision medicine

## Abstract

Describe a novel use for a kinase inhibitor, imatinib, in young children with a known activated somatic mutation in PDGFR‐beta. Two patients with infantile myofibromatosis treated with imatinib. Case description of evaluation, diagnosis and treatment decisions for infantile myfibromatosis of the head and neck. Description of medical therapy for infantile myofibromatosis in these patients. For function threatening myofibromas of a known genotype, in infants, targeted medical therapy is a treatment option.

## Graphical abstract

Tumors associated with infantile myofibromatosis are potentially sensitive to monotherapy with tyrosine kinase inhibitors like imatinib. Such treatment may avoid surgery or chemotherapy, but further study is needed, especially on treatment duration.

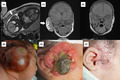

## 1 INTRODUCTION

Infantile myofibromatosis (IM) is a rare proliferative disorder characterized by the growth of soft tissue neoplasms or myofibromas. As the most common soft tissue tumor in children, myofibromas occur in 1/400,000 births.^1^ The associated lesions appear at birth in 60% of cases, with 88% appearing by two years of age. One‐third of cases occur in the head and neck region.^2^ Gain‐of‐function pathogenic variants of the *PDGFRB* gene, coding for the platelet‐derived growth factor receptor β (PDGFR‐β) tyrosine kinase, have been implicated in IM, distinguishing them from other types of fibromatosis. Recently, tyrosine kinase inhibitors such as sorafenib and imatinib have been used in conjunction with traditional chemotherapy and surgery to control an aggressive case of IM.^3^ In this report, we present two cases of IM with head and neck manifestations in which surgery was avoided through treatment with imatinib monotherapy. These patients were recently described as part of a larger series on PDGFRB activating variant spectrum disorder (PAVS). PAVS1 encompasses IM and aneurysms, while PAVS2 includes Penttinen syndrome and Kosaki overgrowth syndrome.^4^

## 2 CASE REPORTS

### 2.1 Patient 1

**FIGURE 1:**
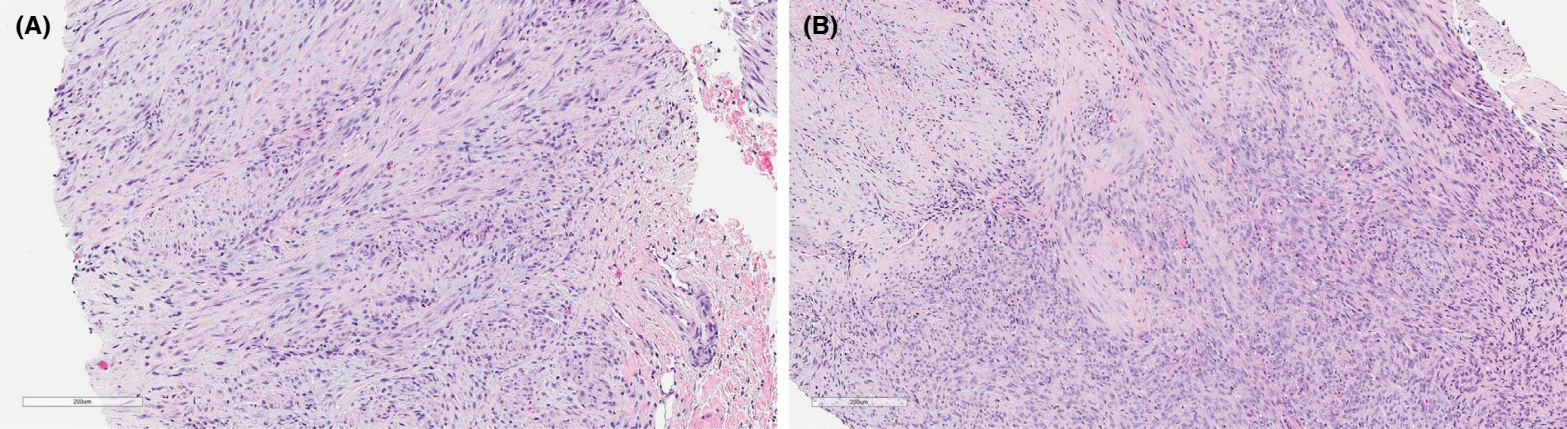
Hematoxylin and eosin‐stained histologic images from the skin biopsy (A, patient 1) and neck mass (B, patient 2). Both masses show broad fascicles of spindled cells with moderate amounts of eosinophilic cytoplasm. The tumor shows classic zonation, with a peripheral hypercellular region merging with central areas containing increased myxohyaline stroma

Patient 1 was born at 38 6/7 weeks gestation to a family with a history of myofibromatosis. He was born with extensive cutaneous nodules that on biopsy were consistent with myofibroma (Figure 1A). Genetic testing of tissue and peripheral blood (UW‐OncoPlex Cancer Gene Panel^5^) revealed activating pathogenic *PDGFRB* variants. Peripheral blood demonstrated p.R561C variant, while tumor tissue demonstrated both p.R561C and p.N666K variants. Magnetic resonance imaging (MRI) at two weeks of age confirmed myofibromas in the facial, neck, chest wall, paraspinous, and extremity soft tissues.

**FIGURE 2:**
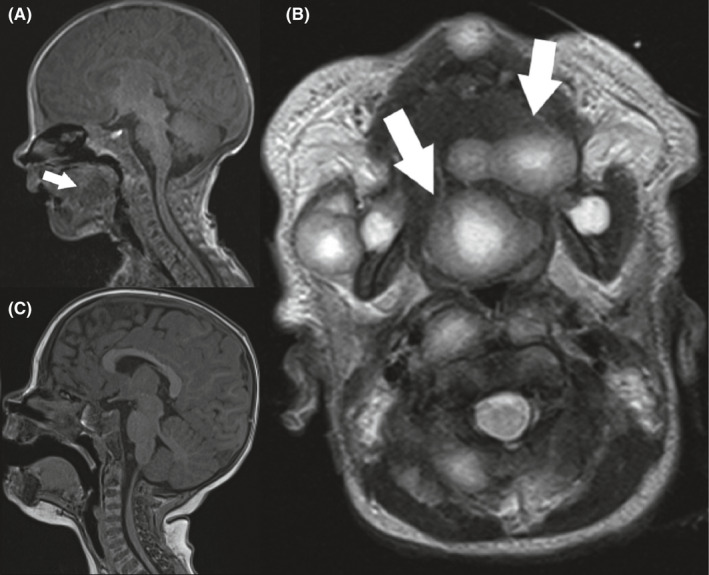
Patient 1 at 2 weeks old demonstrating obstructive, tongue/base of tongue lesion on sagittal T1 MRI (A) and axial T2 (B); arrows designate tongue and base of tongue myofibromas. Sagittal T1 MRI 6 months after cessation of imatinib demonstrating absence of myofibroma (C)

A large tongue myofibroma measuring 1.9 cm was demonstrated on the MRI (Figure 2A, B). As the lesion grew, his symptoms escalated from feeding difficulties to airway obstruction, necessitating continuous positive airway pressure (CPAP) ventilation. Direct laryngoscopy and bronchoscopy revealed airway compromise due to the myofibroma located at the base of tongue (Cormack–Lehane Grade IV laryngeal view), without other airway masses or causes for airway obstruction.

Due to progressive aerodigestive tract compromise at 4 weeks old, imatinib was started (6 mg/kg/day). Subcutaneous and tongue lesions decreased in size, and airway compromise improved. After three weeks of imatinib treatment, all respiratory and oxygen support were weaned off. After a month, he was discharged from the hospital, with the tongue lesion barely palpable. He underwent monthly dose adjustments for weight as an outpatient. Imatinib was discontinued after fourteen weeks, with MRI at that time demonstrating near‐complete resolution of all cutaneous lesions and resolution of the tongue lesion. Over the next nine months of follow‐up of imatinib, there was interval regrowth of the cutaneous and muscular nodules clinically and on MRI, but not of the tongue lesion (Figure 1C). The family elected to observe the other lesions.

### 2.2 Patient 2

**FIGURE 3:**
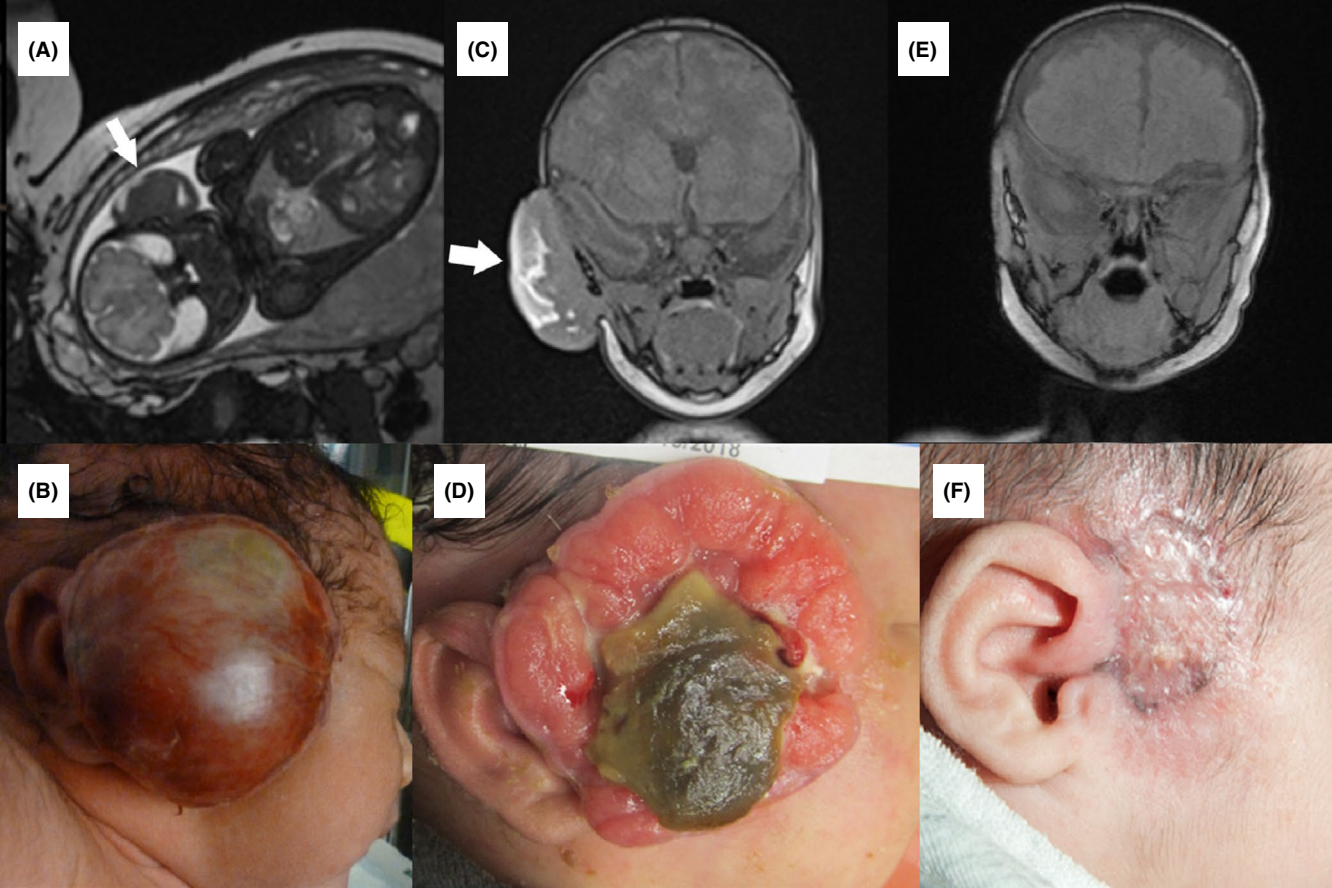
Myofibroma for Patient 2 on fetal MRI (A) and at birth (B). MRI and clinical appearance at one month of age, immediately prior to starting imatinib (C and D, respectively). MRI and clinical appearance after 2 months on imatinib monotherapy (E and F, respectively). Arrows designate facial myofibroma on imaging. Reprinted with permission from Wenger, et al^3^

Patient 2 was born at 35 4/7 weeks via Caesarian section because of a prenatally diagnosed, large solid, partially necrotic right facial mass (Figure 3A, B). Imaging also demonstrated focal skeletal lucencies. Incisional biopsy and debridement of the facial mass were performed on Day six of life. The mass developed a non‐healing ulcerative, necrotic wound requiring wound care and admission for secondary infection (Figure 3C, D). Histologically, the biopsy confirmed myofibroma (Figure 1B). Genetic testing (UW OncoPlex^5^) of peripheral blood was negative for germline changes in *PDGFRB*, but two somatic variants were noted in the tissue sample (p.I564_V572del and p.N666T).

At 35 days old, imatinib was started (6 mg/kg/day). Response was brisk, with flattening and healing of the ulcerated mass over the next 2 months (Figure 3E, F). During that same time, MRI and skeletal survey demonstrated resolution or improvement of soft tissue and bony lesions. The patient remained on therapy for 5 months. During the last two months of treatment, she required fortified formula supplementation due to slowed weight gain.

## 3 DISCUSSION

IM (or PAVS1) can be a devastating disease affecting children less than two years old. Approximately one‐third of cases involve the head and neck and can occur in any subsite, making the disease of considerable importance to otolaryngologists.^2^ Given that many such lesions will regress over time, traditional management includes observation for quiescent lesions, while surgery or chemotherapy has been used for growing myofibromas.^6^, ^7^ In neonates and infants, either surgical excision of large infiltrative head and neck masses or chemotherapy is highly morbid.^8^ In our two patients, the myofibromas were not quiescent and surgical resection would have been extensive and would have probably required repeated procedures, with significant functional consequences.

In these patients, it was thought that targeted medical therapy directed at the causal activated gene variant would have less risk and be more effective than traditional therapies. The treatment plan was supported by in vitro evidence,^9^, ^10^ a prior case of imatinib and sorafenib in combination with traditional chemotherapy,^3^ and experience with other *PDGFRB*‐mediated conditions.^4^ Major surgeries were avoided in both instances. In Patient 1, worsening airway obstruction requiring positive‐pressure ventilation would have ultimately required tracheostomy. In patient 2, the non‐healing ulcerated myofibroma healed and regressed, so that extensive surgery and reconstruction were unnecessary. We have demonstrated that imatinib offers a new and effective therapy for selected IM patients by targeting their known activating *PDGFRB* variants.

Further research is needed to understand optimal treatment duration and risks in this population. It is noteworthy that for Patient 1, improvements in the tongue myofibroma were clinically durable after imatinib cessation, while cutaneous and other soft tissue lesions were not. For Patient 2, long‐term assessment was not possible. Given the dramatic results seen in our patients, imatinib monotherapy should be considered prior to surgery or chemotherapy for patients with activating variants in *PDGFRB*. It is possible that adjunctive use of tyrosine kinase inhibitors will allow for more targeted surgical approach to myofibromas. Adverse events, such as decreased growth velocity or dermatitis, cannot be fully ascertained in this limited study.^4^ These risks warrant further study.

## 4 CONCLUSION

Frequently causing myofibromas in the head and neck, IM can be devastating, but the recognition that it is caused by gain‐of‐function variants in the PDGFR‐β tyrosine kinase receptor has led to a unique opportunity for precision medicine. Recent literature supports the use of imatinib and other tyrosine kinase inhibitors in the treatment of IM. Our experience treating two infants with myofibromas using imatinib monotherapy suggests that targeted treatment of PAVS1 may be a safe and effective to manage these patients, avoiding surgery or chemotherapy. Further research is required to determine optimal treatment duration and potential adverse events for patients on this targeted therapy.

## CONFLICT OF INTEREST

The authors have no relevant financial disclosures or conflicts of interest.

## AUTHOR CONTRIBUTIONS

Prasanth Pattisapu, MD, MPH, originated and edited manuscript. Tara L. Wenger, MD; John P. Dahl, MD, PhD, MBA; Randall A. Bly, MD; Juliana Bonilla‐Velez, MD; Natalie Wu, MD; Anurekha Hall, MD; Erin R. Rudzinski, MD; and Jonathan A. Perkins, DO, cowrote and edited manuscript.

## ETHICAL APPROVAL

Approval for this case report was obtained from the Seattle Children's IRB (STUDY00002158).

## CONSENT

Informed consent from patients' families was obtained for publication.

## HUMAN PARTICIPANTS PROTECTION

Approval for this case report was obtained from the Seattle Children's IRB (STUDY00002158). Written informed consent was obtained from the parents of patients to publish this report in accordance with the journal's patient consent policy.

## Data Availability

Data sharing was not applicable to this article as no datasets were generated or analyzed during the current study.

## References

[1] Coffin CM , Dehner LP . Soft tissue tumors in first year of life: a report of 190 cases. Pediatr Pathol. 1990;10(4):509‐526. doi:10.3109/15513819009067140 2164660

[2] Chung EB , Enzinger FM . Infantile myofibromatosis. Cancer. 1981;48(8):1807‐1818. doi:10.1002/1097-0142(19811015)48:8<1807:aid-cncr2820480818>3.0.co;2-g 7284977

[3] Bidadi B , Watson A , Weigel B , Oliveira A , Kirkham J , Arndt C . Treatment of generalized infantile myofibromatosis with sorafenib and imatinib: a case report. Pediatr Blood Cancer. 2020;67(6):e28288. doi:10.1002/pbc.28288 32307894

[4] Wenger TL , Bly RA , Wu N , et al. Activating variants in PDGFRB result in a spectrum of disorders responsive to imatinib monotherapy. Am J Med Genet A. 2020;182(7):1576‐1591. doi:10.1002/ajmg.a.61615 32500973

[5] Pritchard CC , Salipante SJ , Koehler K , et al. Validation and implementation of targeted capture and sequencing for the detection of actionable mutation, copy number variation, and gene rearrangement in clinical cancer specimens. J Mol Diagn. 2014;16(1):56‐67. doi:10.1016/j.jmoldx.2013.08.004 24189654 PMC3873496

[6] Mashiah J , Hadj‐Rabia S , Dompmartin A , et al. Infantile myofibromatosis: a series of 28 cases. J Am Acad Dermatol. 2014;71(2):264‐270. doi:10.1016/j.jaad.2014.03.035 24894456

[7] Zhao G , Zhu M , Qin C , Liu X , Zhao X . Infantile myofibromatosis: 32 patients and review of literature. J Pediatr Hematol Oncol. 2020;42(8):495‐498. doi:10.1097/MPH.0000000000001603 31764512

[8] Levine E , Fréneaux P , Schleiermacher G , et al. Risk‐adapted therapy for infantile myofibromatosis in children. Pediatr Blood Cancer. 2012;59(1):115‐120. doi:10.1002/pbc.23387 22038698

[9] Arts FA , Chand D , Pecquet C , et al. PDGFRB mutants found in patients with familial infantile myofibromatosis or overgrowth syndrome are oncogenic and sensitive to imatinib. Oncogene. 2016;35(25):3239‐3248. doi:10.1038/onc.2015.383 26455322

[10] Hassan M , Butler E , Wilson R , et al. Novel PDGFRB rearrangement in multifocal infantile myofibromatosis is tumorigenic and sensitive to imatinib. Cold Spring Harb Mol Case Stud. 2019;5(5):a004440. doi:10.1101/mcs.a004440 31645346 PMC6824247

